# Complex Modal Characteristic Analysis of a Tensegrity Robotic Fish’s Body Waves

**DOI:** 10.3390/biomimetics9010006

**Published:** 2023-12-24

**Authors:** Bingxing Chen, Jie Zhang, Qiuxu Meng, Hui Dong, Hongzhou Jiang

**Affiliations:** 1 School of Mechanical Engineering and Automation, Fuzhou University, Fuzhou 350108, China; bingxingchen@fzu.edu.cn (B.C.); 210227168@fzu.edu.cn (J.Z.); 230227053@fzu.edu.cn (Q.M.); 2 School of Mechatronics Engineering, Harbin Institute of Technology, Harbin 150001, China

**Keywords:** tensegrity robotic fish, tensegrity structure, fish body wave, the complex orthogonal decomposition method, traveling index

## Abstract

A bionic robotic fish based on compliant structure can excite the natural modes of vibration, thereby mimicking the body waves of real fish to generate thrust and realize undulate propulsion. The fish body wave is a result of the fish body’s mechanical characteristics interacting with the surrounding fluid. Thoroughly analyzing the complex modal characteristics in such robotic fish contributes to a better understanding of the locomotion behavior, consequently enhancing the swimming performance. Therefore, the complex orthogonal decomposition (COD) method is used in this article. The traveling index is used to quantitatively describe the difference between the real and imaginary modes of the fish body wave. It is defined as the reciprocal of the condition number between the real and imaginary components. After introducing the BCF (body and/or caudal fin) the fish’s body wave curves and the COD method, the structural design and parameter configuration of the tensegrity robotic fish are introduced. The complex modal characteristics of the tensegrity robotic fish and real fish are analyzed. The results show that their traveling indexes are close, with two similar complex mode shapes. Subsequently, the relationship between the traveling index and swimming performance is expressed using indicators reflecting linear correlation (correlation coefficient ( $R_{c}$ ) and *p* value). Based on this correlation, a preliminary optimization strategy for the traveling index is proposed, with the potential to improve the swimming performance of the robotic fish.

## 1. Introduction

Fish have attracted the attention of many scholars’ because of their excellent swimming characteristics. The related field of bionics is a hot spot of scientific research [1,2,3,4,5,6]. The propulsion mechanism of fish is a crucial aspect of this study. Breder [7] categorizes propulsion mechanisms into two modes: BCF (body and/or caudal fin) mode and MPF (median and/or paired fin) mode. BCF fish generate body waves through the undulatory body and oscillatory caudal fin, which is the result of the mechanical characteristics of the fish body interacting with the surrounding fluid [8]. The viscoelastic properties of fish muscles play a significant role in the bending deformation of their bodies [9,10].

Numerous scholars have advanced dynamic analyses of fish, for example, conceptualizing their bodies as deformable viscoelastic beams immersed in water [9,11,12,13]. The fish body wave is governed by the stiffness component and the viscous damping component (including fluid and internal damping) [14]. Fluid damping primarily arises from the interaction between the fish’s body and the surrounding fluid. Internal damping is primarily attributed to the complex internal anatomy of the fish body, consisting of joints and muscles. Following dynamic analysis, this swimming model assumes the form of a wave equation. Fish locomotion involves the forced vibration of the viscoelastic body within a fluid environment [15,16]. Therefore, the fish body wave may originate from vibration mode excitation, and its essence is the complex mode shapes corresponding to fish body vibration [17].

Grounded in this perspective, Feeny [17] proposed the complex orthogonal decomposition (COD) method, which decomposes fish body waves into two components: pure standing waves and pure traveling waves. This method can extract the modes and modal coordinates and estimate fish body wave parameters such as frequency, wavelength, and wave velocity to further quantify fish locomotion. COD is a generalization of the well-known proper orthogonal decomposition (POD). POD, similar to singular-value decomposition (SVD) and principal component analysis (PCA), is a tool used to extract modes that optimize the signal energy distribution in a set of measured time series [18]. POD is particularly useful in extracting standing components but is less suited for decomposition of non-standing components. COD fills this gap. Therefore, COD provides an analysis tool to extract modal information from fish body waves.

Feeny [18] performed a modal analysis of whiting and analyzed the complex modal characteristics of amputated and non-amputated fishes using the traveling index. Cui [19] employed the COD method to analyze biological data of the body waves of over 80 fish. Through this analysis, the locomotion of BCF fish can be broadly classified into three categories by the traveling index: the standing-wave form, the mixture-wave form, and the traveling-wave form. Moreover, Cui et al. [20] analyzed the midline motion of anguilliform fish through dynamic modeling and the COD method, delving into the correlations between the traveling index, body stiffness, and tail-beat frequency.

Fish body motion with a high traveling index consistently directs the flow towards the wake with a direction opposing the body motion, creating thrust through momentum flux [21]. Muller et al. [22] explained that this may occur because the protovortices created near the head continue to grow, adding momentum to the wake when shed by the tail. In the case of a low traveling index, the predominant motion is the standing wave, which displaces the surrounding fluid laterally. Its momentum flux typically occurs in the lateral direction, potentially impacting swimming efficiency [23]. Furthermore, Anastasiadis and Ijspeert et al. [23] pointed out that a higher traveling index can reduce the cost of transport (COT) of robotic fish. The prerequisite to achieving high efficiency is the adoption of pure traveling wave-like body undulations.

BCF bionic robotic fish usually mimic the fish body waves of natural fish to achieve efficient locomotion [24,25,26,27,28,29,30]. Therefore, the COD method can be employed to analyze the complex modal characteristics of both real fish and robotic fish. Generally speaking, there are two main methods for mimicking the body wave motion of BCF fish: the joint motion control method and the vibration-mode excitation method. The former generally adopts super-redundant series or series-parallel, multijoint rigid discrete mechanisms as the fish spine and mimics the fish body wave by individually controlling each joint [31,32,33,34]. Such robotic fish require preplanning of the movement of each joint. Therefore, they struggle to effectively respond to the dynamically changing water vortices during swimming and cannot reproduce the fish’s passive attributes and its dynamic interactions with the environment [35]. However, due to the independent control of each joint, there exists a certain advantage in achieving high maneuverability. For example, the multijoint robotic dolphin developed by Yu et al. [36,37] has made outstanding progress in the jumping motion. Zheng et al. [38,39,40] developed a variety of cable-driven robotic fish, which achieved 1.37 BL/s and a turning rate of 457°/s through central pattern generator control.

Robotic fish using the second method generally employ single-point sinusoidal excitation to excite the compliant body’s vibration modes, thus mimicking fish body waves to achieve efficient undulatory propulsion [9]. The study of bio-inspired robotic fish can be traced back to the work of McHenry and Long [41] in the 1990s. They developed a compliant robotic fish using viscoelastic materials. Subsequently, Alvarado [9] used a driver to excite the vibration modes of compliant mechanisms to mimic fish body waves, thus achieving undulatory propulsion. In previous research [42], we developed a freely swimming tensegrity robotic fish, TenFiBot-*I*, with the fish body consisting of a tensegrity structure. The tensegrity structure typically consists of compression elements (such as rigid bodies and rods) and continuous-tension elements [43]. Compression elements only experience compression, while tension elements only experience tension. The overall integrity and stability of the entire structure can be achieved through the use of a cable tension network [44]. The actuation methods for tensegrity robotics include pneumatic actuation, intelligent metal actuation, cable actuation, etc. [45]. Cable actuation is widely used and achieved by changing the length and force of the active tension element. Our robotic fish follows this approach. Experiments show that the robotic fish can excite C-shaped and S-shaped vibration modes through single-point excitation [42].

Unlike the joint motion control method, the fish body waves of these robotic fish are similar to those of real fish. However, current research primarily focuses on the design of physical prototypes and the control of vibration-mode excitation, while studies on the complex modal characteristics of fish body waves remain limited. Indeed, exploring the relationship between fish body wave characteristics and swimming performance is an important academic direction [13,46,47,48,49]. Therefore, in-depth exploration in this field can contribute to our understanding the propulsion performance of robotic fish and aid in optimizing iterations.

To address this issue, based on our previous research [42], in this paper, we analyze the complex modal characteristics of tensegrity robotic fish. The remaining sections are outlined as follows. In Section 2, the definition of fish body wave is introduced. An analysis of the body waves of four BCF fish is conducted, specifically anguilliform, subcarangiform, carangiform, and thunniform fish. The COD method is introduced. In Section 3, the experimental setup of the tensegrity robotic fish is presented, including the hardware configuration and the design of the experimental swimming platform. In Section 4, the experimental results are analyzed. The fish body waves and the decomposition of a robotic fish with three driving frequencies are reported. The similarities and differences in the complex modal characteristics between robotic fish and real fish are compared. Then, we analyze the relationship between the traveling index and swimming performance, including driving amplitude, driving frequency, swimming velocity, tail amplitude, stride length, and Strouhal number. The impacts stemming from different drive amplitudes are assessed. A preliminary optimization strategy for the traveling index is proposed. Section 5 summarizes the entire article.

## 2. Complex Modal Characteristic Analysis of Fish Body Wave

### 2.1. Fish Body Wave Curve

**Figure 1:**
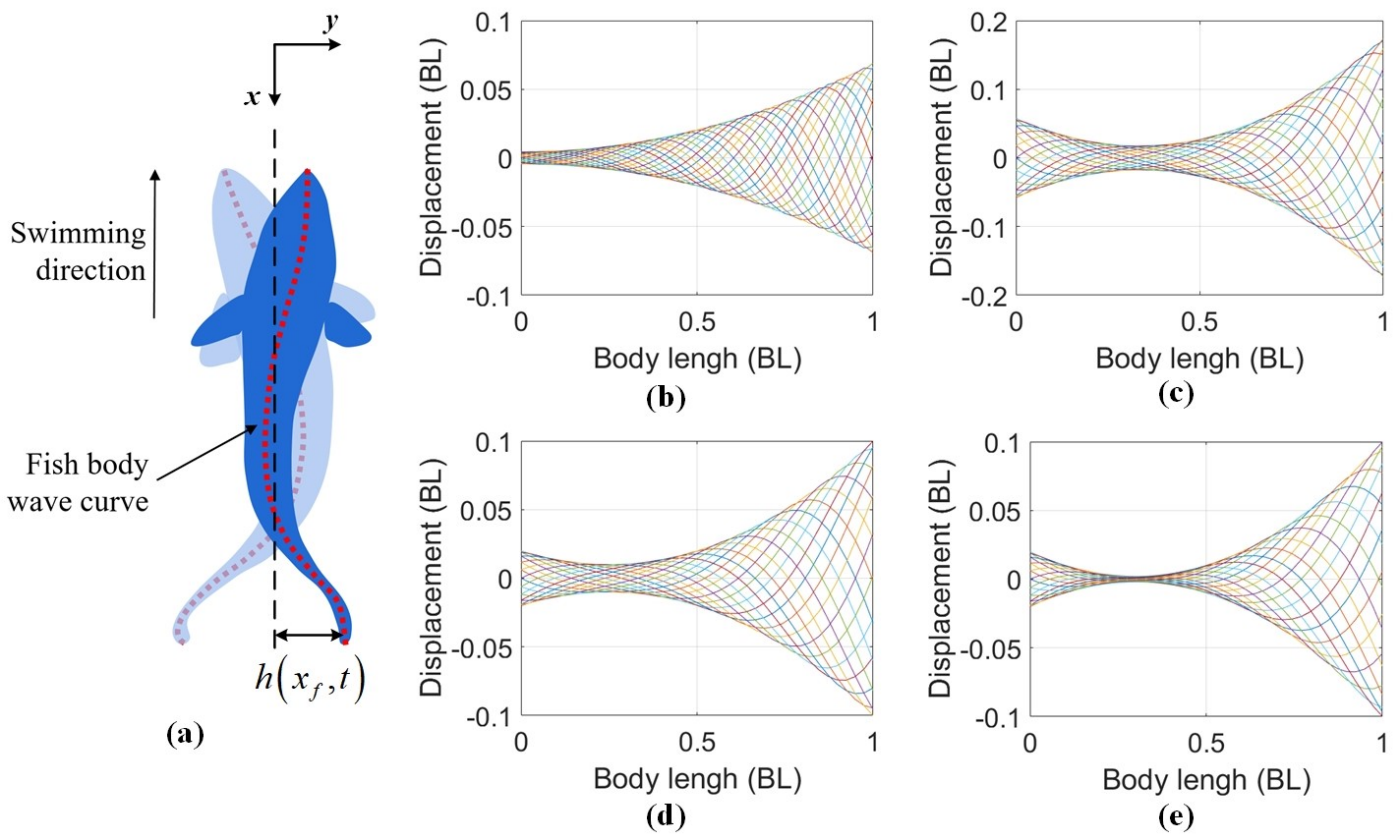
Fish body wave curve: (**a**) Diagram of the fish body wave curve. The blue area is the outline of a BCF fish, and the red line segment is the fish body wave curve. (**b**) Fish body wave curves of anguilliform fish [16]. (**c**) Fish body wave curves of subcarangiform fish [50]. (**d**) Fish body wave curves of carangiform fish [51,52]. (**e**) Fish body wave curves of thunniform fish [9,53]. Curves of different colors represent the body midline displacement of fish at 20 equally spaced time intervals during a single tail beat cycle.

BCF fish generate body waves to effectively transfer momentum to the wake through increased water velocity. The fish’s bending spine curve is often referred to as the fish body wave curve (Figure 1). Based on biological data [9], the fish body wave ( $h\left(x_{f},t\right)$ ) can be fitted as

(1) $$h\left(x_{f},t\right)=H\left(x_{f}\right)\sin\left(\omega_{f}t+k_{f}x_{f}\right)$$

(2) $$H\left(x_{f}\right)=a_{1}+a_{2}x_{f}+a_{3}x_{f}^{2}$$

where $H\left(x_{f}\right)$ is the envelope equation of fish body wave; $a_{1}$ , $a_{2}$ , and $a_{3}$ are envelope coefficients; $k_{f}$ is the wave number; $\omega_{f}$ is the undulation frequency; and $x_{f}$ is the position in the body length direction, measured from fish’s nose tip ( $x_{f}$ = 0) towards the tail, with the same direction along the *x* axis.

BCF fish can be classified into four categories: anguilliform, subcarangiform, carangiform, and thunniform [54]. Figure 1 shows their normalized body wave curves in one cycle of steady-state swimming, with the fish position in units of body length (BL). In Figure 1, the fish’s nose tip is at 0 BL in the fish body position, and the tail tip is at 1 BL. Various parameters, such as amplitude and curvature, can be obtained based on these values. Amplitude refers to the maximum displacement achieved by the fish body in the vertical forward direction during swimming, including the head amplitude and the tail amplitude. The amplitudes vary among BCF fish, but the maximum amplitude consistently occurs at the tail [30].

Curvature indicates the degree of bending in a specific part of the fish’s body. Greater curvature implies more pronounced bending. The maximum curvature always occurs posteriorly, beyond the caudal peduncle [30]. While these parameters directly reflect the swimming state of the robotic fish, their relationships are intricate and challenging to employ in analysis of the complex modal characteristics. Therefore, the COD method is introduced in the next section to evaluate the complex modal characteristics.

### 2.2. The Complex Orthogonal Decomposition Method of Fish Body Wave

The kinematics analysis of fish swimming using the traveling wave is a hot topic in the fields of swimming dynamics and fluid mechanics [55,56,57]. In the field of bionic robotic fish, scholars have experimentally observed that robotic fish performs better [21,22,23,58,59] when the traveling wave is the dominant component of the fish body wave. However, it is challenging to analyze the traveling wave and standing wave. Feeny [17] proposed the COD method to solve this problem, which is a general form of proper orthogonal decomposition (POD). It can analyze the undulatory motion of insects, the vibrations of beams, and the fish body wave of natural fish and robotic fish [60,61]. The fish body wave is decomposed into traveling and standing waves. The steps are as follows.

By numerically discretizing the fish body wave curve of the robotic fish, the lateral displacement matrix ( $Y_{M\times N}$ ) of the fish body is obtained, where $y_{m}\left(t_{n}\right)$ is the element in the *m*th row and *n*th column in the matrix, which represents the lateral displacement of the $x_{m}$ position at the $t_{n}$ moment.

(3) $$\left\{\begin{array}{c} Y_{M\times N}={\left[y_{1},y_{2},\ldots ,y_{M}\right]}^{T}\\ y_{j}={\left[y_{j}\left(t_{1}\right),y_{j}\left(t_{2}\right),\ldots ,y_{j}\left(t_{N}\right)\right]}^{T} \end{array}\right.$$

where $x_{m}=mL/M$ represents the marker points on the robotic fish, evenly distributed along the axial length *L* of the fish body. The axial length (*L*) of the fish body is normalized, and the unit is BL (body length). The time ( $t_{n}=nT/N$ ) is equally distributed within the unit oscillation period (*T*).

Then, the matrix ( $Y_{M\times N}$ ) is transformed into the complex form ( $Z_{M\times N}$ ) by Hilbert transform, which can be used for complex modal analysis.

(4) Z=Y+iH(Y)

where $i=\sqrt{-1}$ is the imaginary unit. In the COD method, it is necessary to solve the characteristic equation containing the fish body wave motion information, that is,

(5) Rv=λv

The matrix (*R*) is a complex coefficient matrix that records the lateral displacement of the fish body at different times. It is a Hermitian matrix of size m×m . The eigenvectors (*v*) of *R* are called “complex orthogonal modes” (COMs) and indicate mode shapes that represent the characteristic movement of the fish [18]. The eigenvalue $\lambda \approx \frac{M}{L}D_{j}$ is the “complex orthogonal values” (COVs) in units of length squared, and $D_{j}$ is the mean square amplitude of the fish body [17]. The largest COV corresponds to the dominant wave form of the swimming fish. The matrix (*R*) can be defined as

(6) $$R=\frac{1}{N}Z{\bar{Z}}^{T}$$

The feature vector can be expressed as

(7) v=c+id

where *c* and *d* represent two different mode shapes. The fish body wave signal can be regarded as a continuous transition form between two different mode shapes (*c* and *d*). The correlation between them indicates a mixed relationship between traveling waves and standing waves. Therefore, the main motion corresponding to the main mode of the fish body’s lateral motion is

(8) $$z_{1}\left(t\right)=e^{i\omega t}\left(c+id\right)$$

where ω is the oscillation frequency. The lateral movement of a robotic fish body can be approximated by the main movement, which can be expressed as

(9) h(t)≈Re(z1(t))=ccosωt−dsinωt

The fish body’s lateral movement varies periodically between vibration modes *c* and *d*. When the real and imaginary components of the modes are equal, the lateral movement is in the form of a standing wave. Displacements at different points on the fish body do not reach the maximum values simultaneously. Conversely, when the real and imaginary components are unequal, the lateral movement exhibits the form of a traveling wave. The traveling index is written as

(10) $$\alpha =\frac{1}{cond(\left[c,d\right])}$$

The term ”traveling index” originates from Feeny’s work [18], defined as the reciprocal of the condition number between the real and imaginary components of the complex mode. The condition number ( cond ) is defined as the product of the matrix norm and the norm of the inverse matrix, which is $cond\left(\left[c,d\right]\right)=∥\left[c,d\right]∥\cdot ∥{\left[c,d\right]}^{-1}∥$ . Many studies have used the traveling index to analyze fish body waves [19,62,63]. The relative sizes and degrees of independence of *c* and *d* dictate the “amounts” of standing and traveling in the wave. Pure traveling waves have orthogonal components of the same magnitude, leading to a condition number of 1 and, hence, a traveling index of 1. Deviations, either in the magnitudes of the component vectors or the directions, lead to larger condition numbers. Vectors lying in the same direction (completely dependent) or of greatly differing magnitudes have large condition numbers and, hence, small traveling indexes. As the traveling index approaches zero, there is essentially one independent vector, representing purely standing motion.

## 3. Experimental Program of the Tensegrity Robotic Fish

**Figure 2:**
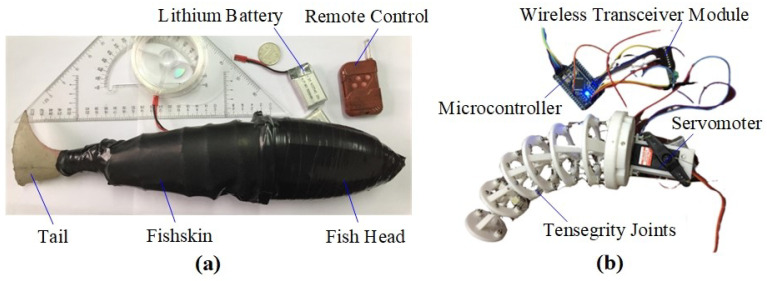
Structural diagram of the tensegrity robotic fish: (**a**) physical picture; (**b**) internal structure.

The structural diagram of the tensegrity robotic fish, TenFiBot-*I*, is illustrated in Figure 2. It consists of three components, including a rigid fish head, a fish body composed of six tensegrity joints, and a compliant tail fin. In the swimming experiment, the driving amplitudes (swing angle of the servo motor) are 36°, 45°, 54°, and 63°. The servo motor has a position-feedback potentiometer and contains a closed-loop control system. The microcontroller reads the current angular position of the output shaft of the servo motor through the position-feedback potentiometer and compares it with the set position target. According to the phase deviation, the actual position of the output shaft is adjusted to match the target position, forming a closed-loop feedback and, lastly, outputting an accurate position.

**Figure 3:**
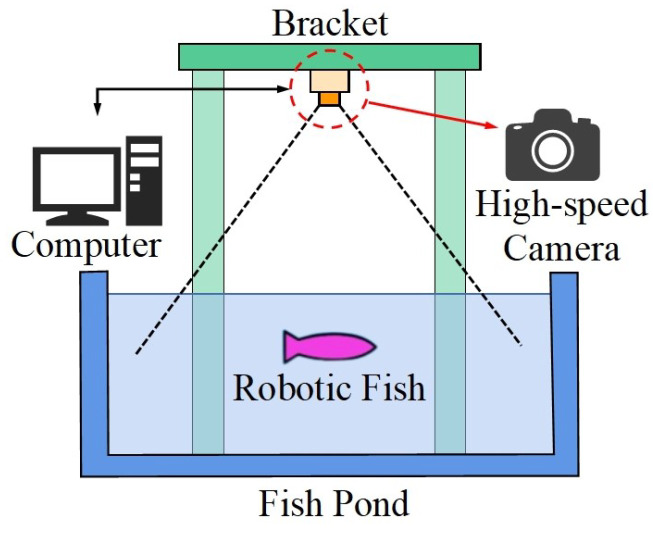
Experimental platform, including a computer, a high-speed camera, a bracket, and a fish pond.

The experimental platform is shown in Figure 3, including a computer, a high-speed camera, a bracket, and a fish pond. The robotic fish was put into a still-water pool for free swimming. Its swimming process was filmed with a camera fixed above the bracket, and the swimming video was stored in a computer. The fish skin is made black to facilitate subsequent image processing. More detailed experimental settings and dimensions can be found in our previous article, and videos of the robotic fish swimming, as well as code analysis, are available in the supplementary material [42].

The tensegrity robotic fish may produce a yaw angle when swimming freely, that is, it cannot move along a straight line. This creates trouble in extracting and analyzing the fish body wave curves. Therefore, the midline reconstruction method [64] is used to resolve the location deviation problem of fish body wave curves. The fish body waves can be deconstructed into two components: periodic components and secular components. The periodic components are represented by the Fourier series, while the secular components are represented by the term of velocities and accelerations of discrete points of the fish body. The periodic components represent the lateral movement of the fish body. The secular components represent the axial motion, which is the location deviation of the fish body, that is, the positional movement of the fish body along the axial direction. In the midline reconstruction, the location deviation problem can be solved by removing the secular component terms and retaining the periodic component terms. The reconstructed fish body wave can be regarded as the generated fish body wave when the fish body is stationary. It can be used to analyze the complex modal characteristics of the robotic fish using the COD method.

## 4. Analysis of Experimental Results

### 4.1. Comparison of the Fish Body Wave’s Complex Modal Characteristics between the Tensegrity Robotic Fish and Real Fish

In our previous study [42], we developed a tensegrity robotic fish named TenFiBot-*I*. The effectiveness of fish body construction using tensegrity joints was validated, and the swimming characteristics were measured. The experimental results demonstrated that the tensegrity joints could enhance mechanical efficiency, leading to some swimming characteristics approaching those of real fish. Subsequently, in this paper, we use the COD method to acquire fish body wave data from TenFiBot-*I* and real fish. A comparative analysis is conducted to contrast their complex modal characteristics.

According to the previous description, we conducted a complex modal characteristic analysis for the fish body’s periodic lateral movement. COVs are used to demonstrate modal dominance. The largest COV corresponds to the dominant wave form of the robotic fish and the swimming fish, that is, the main mode [60]. For the tensegrity robotic fish, at 1.2 Hz, the primary COV value is 0.06412 BL^2^, followed by 0.00054 BL^2^ and 0.00014 BL^2^. The rest of the COVs are below $10^{-17}$ BL^2^. As such, the primary mode dominates, accounting for 98.5% of the signal energy. When the driving frequency is 1.7 Hz, the primary COV value of the robotic fish is 0.04962 BL^2^, followed by 0.00172 BL^2^ and 0.00008 BL^2^. The remaining COVs are also below $10^{-17}$ BL^2^. The primary mode becomes the dominant mode, accounting for 96.5% of the signal energy. Finally, when the driving frequency of the robotic fish is 2.9 Hz, the primary COV value is 0.01687 BL^2^, followed by 0.00106 BL^2^, 0.00015 BL^2^, and smaller values. The primary mode dominates, accounting for 93.31% of the signal energy. Under different driving frequencies, the primary modes of the robotic fish’s body waves are dominant, and their complex modal characteristics are analyzed.

According to the COD method, when the driving amplitude is 54°, the robotic fish’s body waves are decomposed into the standing and traveling components at different frequencies. The selected frequencies correspond to 1.2 Hz, which aligns with the maximum step length, and 1.7 Hz, corresponding to the maximum swimming velocity [42]. Additionally, 2.9 Hz is chosen to illustrate higher-order vibration modes.

**Figure 4:**
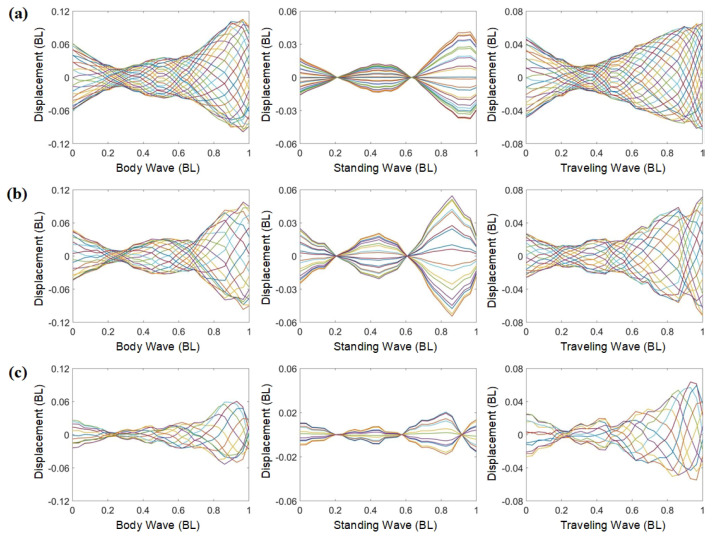
Fish body waves and the standing and traveling components of the tensegrity robotic fish at different driving frequencies: (**a**) driving frequency of 1.2 Hz; (**b**) driving frequency of 1.7 Hz; (**c**) driving frequency of 2.9 Hz. Curves of different colors represent body midline displacements of the robotic fish at equally spaced time intervals during a single tail beat cycle.

The fish body waves and their traveling and standing components at three driving frequencies are shown in Figure 4. At a driving frequency of 1.2 Hz (Figure 4a), the head amplitude of the robotic fish is approximately 0.12 BL, with the maximum amplitude occurring at the tail, around 0.22 BL. The maximum amplitudes of the standing and traveling components also occur at the tail and are 0.08 BL and 0.14 BL, respectively. The traveling components are consistent with the change in the fish body waves; both decrease first, then increase. It reaches the lowest value around 0.3 BL from the fish head and its highest value near the tail. At this point, the traveling index is approximately 0.6, with the traveling component surpassing the standing component. The traveling wave motion is the dominant motion of the robotic fish. At a driving frequency of 1.7 Hz (Figure 4b), the trend in fish body waves remains close to 1.2 Hz, although the overall amplitude slightly decreases. The proportion of the standing component increases, with its maximum amplitude reaching 0.11 BL, leading to a reduction in the traveling component. The traveling index is around 0.45, and the standing component exceeds the traveling component. When the driving frequency is 2.9 Hz (Figure 4c), a notable reduction in amplitude is observed. The head amplitude is merely 0.06 BL, and the maximum amplitude reaches 0.12 BL. The traveling index is at around 0.6—the same as at the driving frequency of 1.2 Hz. Similarly, at this frequency, the dominant motion of the robotic fish is characterized by traveling wave motion.

**Figure 5:**
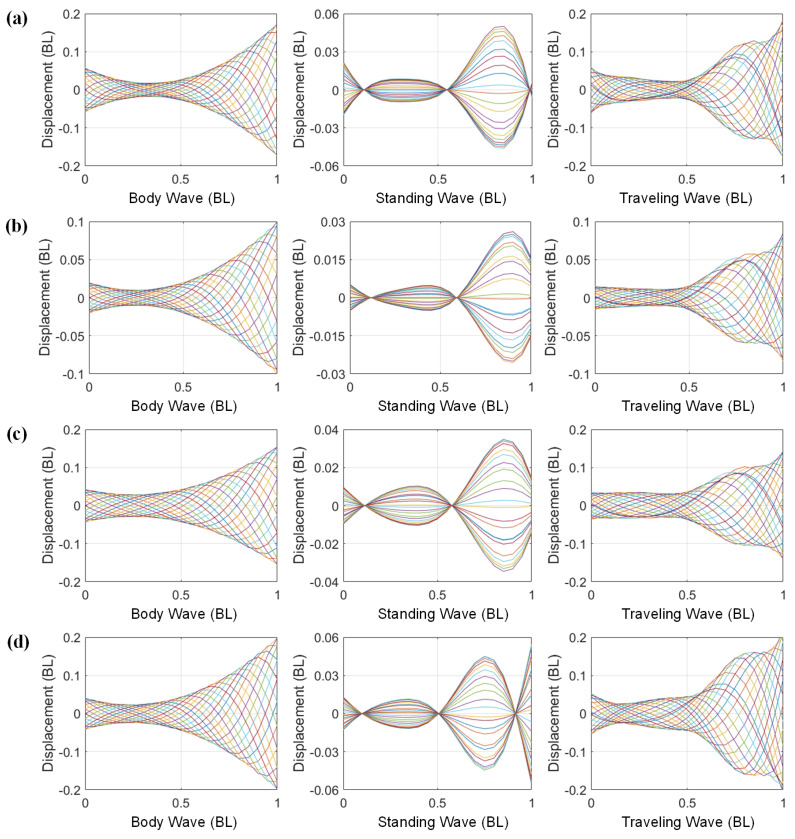
Analysis of complex modal characteristics of different fishes: (**a**) goldfish with the a traveling index of 0.6378 [50]; (**b**) mackerel with a traveling index of 0.6742 [52]; (**c**) whiting with a traveling index of 0.7224 [65]; (**d**) zebrafish larvae with a traveling index of 0.6851 [66]. Curves of different colors represent body midline displacements of fish at 20 equally spaced time intervals during a single tail beat cycle.

The bionic inspiration for the tensegrity robotic fish is derived from subcarangiform and carangiform fishes, which exhibit similar swimming characteristics. To compare the complex modal characteristics between them and the robotic fish, we select several fishes for illustrative analysis (Figure 5), including goldfish, mackerel, whiting, and zebrafish larvae. Their primary COV values are 0.0867 BL^2^, 0.0299 BL^2^, 0.08522 BL^2^, and 0.12798 BL^2^, respectively, representing 99.87%, 99.86%, 99.85%, and 99.86% of the signal energy. Similar to the tensegrity robotic fish, the primary mode predominates as the dominant mode. Next, the complex modal characteristics of the body waves of real fish are analyzed.

Figure 5a–d show the standing and traveling components of goldfish, mackerel, whiting, and zebrafish larvae, respectively. Although the fish body waves differ among fishes, their dominant components are all traveling waves. The amplitude decreases initially, then increases. The minimum amplitude occurs at around one-third of the body length from the head, while the maximum amplitude corresponds to the tail amplitude. The robotic fish exhibits similarities to these fishes. Moreover, the standing components of mackerel and whiting exhibit two stagnation points, whereas the zebrafish larvae display three stagnation points, indicating differences in their vibration modes.

The tensegrity robotic fish similarly exhibits the two complex modal fish body waves observed in subcarangiform and carangiform fishes. By comparing Figure 4a–c, it is clear that the modal shapes at the frequency of 2.9 Hz differ from the other two cases. Figure 4c reveals that the standing component possesses three stagnation points, similar to zebrafish larvae (Figure 5d). In contrast, Figure 4a,b display standing components similar to those of mackerel (Figure 5b) and whiting (Figure 5c), with only two stagnation points. The fish body waves at the 2.9 Hz frequency correspond to the higher-order vibration modes. In contrast, the modal shapes at the other two frequencies are associated with lower-order vibration modes. This illustrates that the robotic fish can effectively mimic the body waves of real fish through various vibration modes. Its decomposed traveling and standing components also correspond to those of the subcarangiform and carangiform fishes.

### 4.2. Relationship between the Traveling Index and Swimming Performance

In this section, we use the COD method to obtain the traveling indexes under different driving amplitudes and frequencies. Then, the relationship between the traveling index and swimming performance is discussed. Initially, the boxplot command in MATLAB is utilized to eliminate outliers from the dataset. Outliers refer to data points in a dataset that are significantly different or abnormal compared to other data points. They are values that are more than 1.5 times the interquartile range from the bottom or top of the box [46]. Then, the data are subjected to linear fitting using the least squares method, and the correlation between parameters is assessed using the correlation coefficient ( $R_{c}$ ) and *p* value.

**Figure 6:**
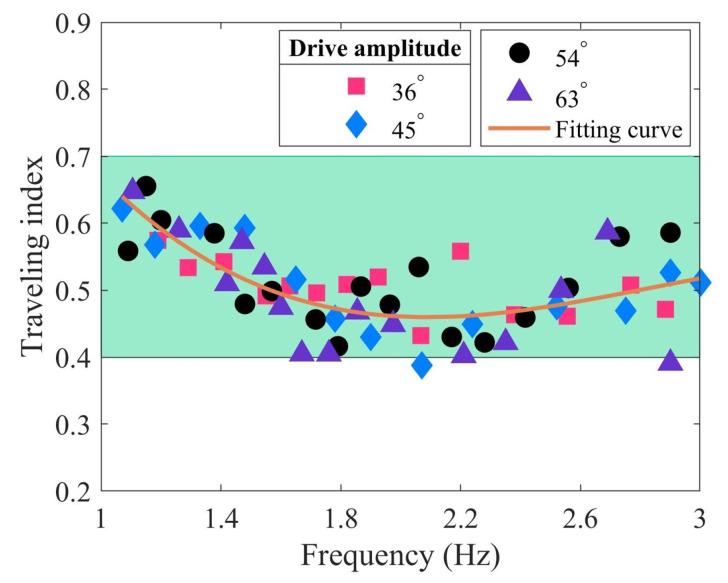
The traveling index of the tensegrity robotic fish at varying driving frequencies.

Figure 6 illustrates the range of the traveling index, which falls between approximately 0.4 and 0.7 (green area). As the driving frequency increases, the traveling index decreases initially, then increases slowly. Based on biological data, the traveling index for subcarangiform and carangiform fishes ranges from approximately 0.52 to 0.78 [19]. For whiting, the traveling index is 0.483 in the body coordinate system and 0.5209 in the inertial coordinate system [18]. The traveling index of TenFiBot-*I* closely approximates these data but still presents distinctions. The primary reason might be that the robotic fish cannot adjust its body stiffness while swimming, thereby failing to adapt to the fluid environment. In contrast, real fish can regulate their bending stiffness, allowing them to achieve improved swimming performance. This may be attributed to the alignment between muscle-driven body stiffness and tail-beat frequency [20,67,68,69].

Table 1 shows the correlation coefficient ( $R_{c}$ ) and *p* values between the swimming performance and the traveling indexes of the robotic fish. $R_{c}$ (Pearson correlation coefficient) is used to measure the degree of correlation between two variables, and the value range is [−1, 1] [70]. When the absolute value of $R_{c}$ is closer to 1, the correlation between variables is very high. The *p* value is used to describe the probability of an event occurring [71]. If the *p* value is less than 0.01, it is considered to pass the significance test, indicating that the analysis is statistically significant.

**Figure 7:**
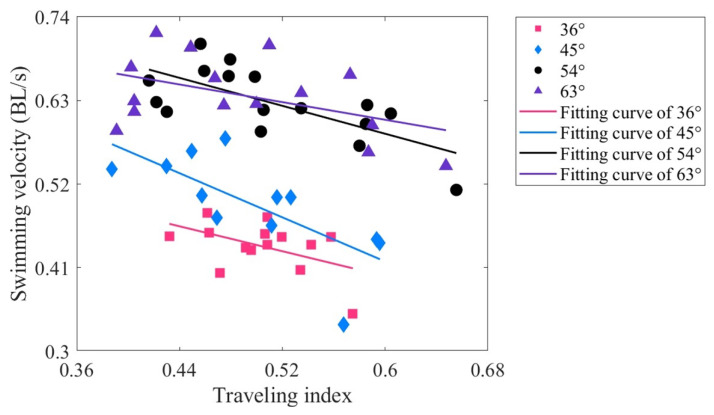
Relationship between the swimming velocity and the traveling index at varying drive amplitudes.

Figure 7 presents the relationship between the swimming velocity and traveling index of the robotic fish at varying drive amplitudes. The swimming velocity range is between 0.3 and 0.74 BL/s. Under different traveling indexes, the swimming velocities of 54° and 63° are higher, with the highest velocity being about 0.72 BL/s. The swimming velocities of 36° and 45° are lower, approximately below 0.58 BL/s. The correlation coefficients between the swimming velocity and the traveling index at four driving amplitudes are −0.487, −0.717, −0.700, and −0.463, respectively. Overall, there is a moderate or high negative correlation, which implies that as the traveling index increases, the swimming velocity decreases. Except for 45°, all *p* values are less than 0.01, indicating a significant linear relationship between swimming velocity and traveling index, and the analysis holds statistical significance. The relatively large *p* value at 45° may be attributed to experimental measurement errors or the small sample size.

**Figure 8:**
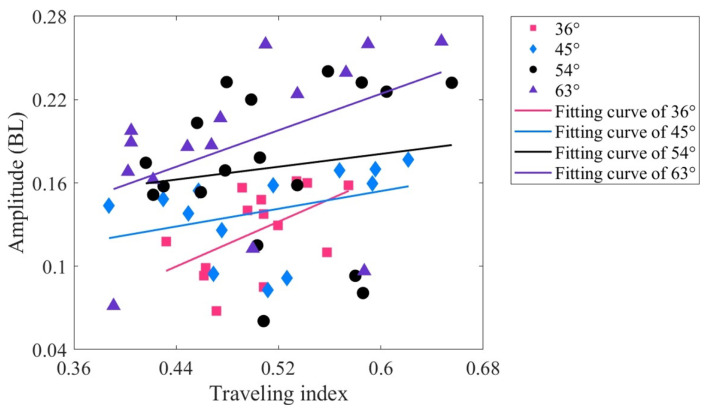
Relationship between the tail amplitude and traveling index at varying drive amplitudes.

The tail amplitude is defined as a peak-to-peak distance of midline motions at the tail tip [72]. Figure 8 shows the relationship between the tail amplitude and the traveling index at varying drive amplitudes. The variation range of the tail amplitude is approximately 0.04–0.28 BL, with the maximum tail amplitude corresponding to a driving amplitude of 63°. When the driving amplitude is 36°, the correlation coefficient between the tail amplitude and the traveling index is 0.519 (*p* < 0.01), indicating a moderately positive correlation. For driving amplitudes of 45° and 63°, the correlation coefficients are 0.360 and 0.452, respectively (both *p* values less than 0.01), indicating a low positive correlation. However, at a driving amplitude of 54°, the correlation coefficient is only 0.141 (*p* < 0.01), indicating that there is almost no relationship at this driving amplitude. It can also be seen from Figure 8 that the data at 54° are relatively discrete. Overall, the correlation between tail amplitude and traveling index is low, and its underlying mechanism needs to be studied.

**Figure 9:**
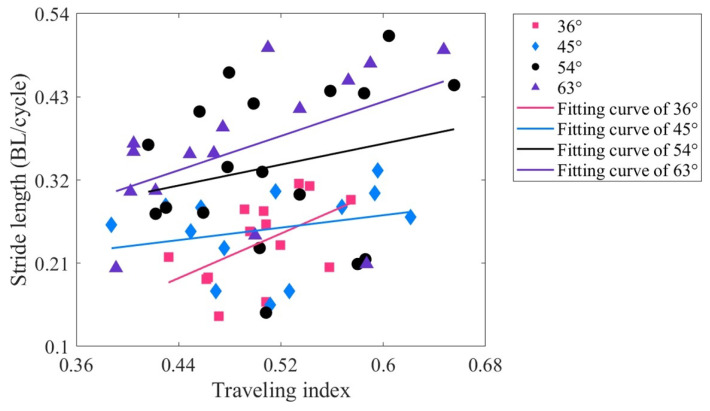
Relationship between the stride length and traveling index at varying drive amplitudes.

The stride length ( $U^{*}$ ) is the swimming distance of a robotic fish in one amplitude cycle, which can be used to reflect the swimming performance [53]. It can be written as $U^{*}=U/f$ , where *U* is the steady-state swimming velocity, and *f* is the driving frequency. Figure 9 illustrates the relationship between the stride length and traveling index at varying drive amplitudes. Upon comparing Figure 8 and Figure 9, it is evident that the trends of the fitted lines for stride length and tail amplitude are roughly similar. When the driving amplitude is 36°, there is a moderately positive correlation between stride length and traveling index ( $R_{c}$ = 0.522, *p* < 0.01). The remaining driving amplitudes are of low correlation. This implies that at lower driving amplitudes, increasing the traveling index has the potential to increase both the tail amplitude and the stride length.

**Figure 10:**
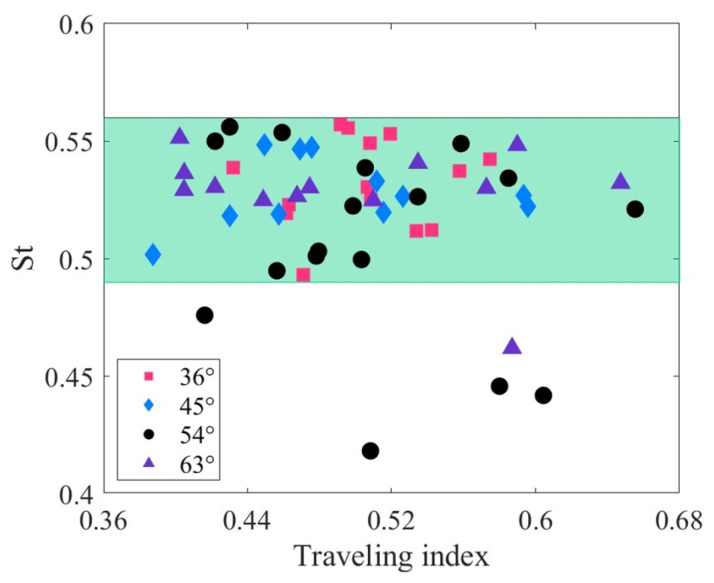
Relationship between the Strouhal number and traveling index at varying drive amplitudes.

Figure 10 illustrates the relationship between the Strouhal number and the traveling index at varying drive amplitudes. The Strouhal number can be written as St=fA/U , where *A* is the wake width (usually approximated as the peak-to-peak tail-beat amplitude [73]). Biological studies show that the optimal Strouhal number for fish swimming is not constant. It depends on the fish’s velocity and the Reynolds number of the fluid [74]. According to Figure 6, the traveling index of the robotic fish ranges from approximately 0.4 to 0.7. The corresponding Strouhal numbers, however, are concentrated within a narrow range of approximately 0.49 to 0.56 (green area). Under different driving amplitudes, the correlation coefficients between the Strouhal number and the traveling index are all less than 0.3, indicating that there is essentially no significant relationship between them. Similar to the robotic fish, various fishes, such as anguilliform, subcarangiform, carangiform, and thunniform, exhibit a considerable range of traveling indexes, ranging from approximately 0.36 to 0.9. These fishes also correspond to an optimized Strouhal number interval of 0.25 to 0.35 [66,75,76,77,78,79]. The independence of the Strouhal number from the traveling index of the tensegrity robotic fish is consistent with that of natural fish.

In conclusion, the relationship between swimming velocity and traveling index demonstrates a high negative correlation. The tail amplitude and stride length exhibit a relatively low positive correlation with the traveling index. After reasonable parameter configuration, the tensegrity robotic fish has significant potential to enhance swimming velocity, tail amplitude, and stride length by adjusting the traveling index. The Strouhal number is unrelated to the traveling index, but maintaining the robot fish’s Strouhal number within an optimal range could be a focal point for future research.

**Figure 11:**
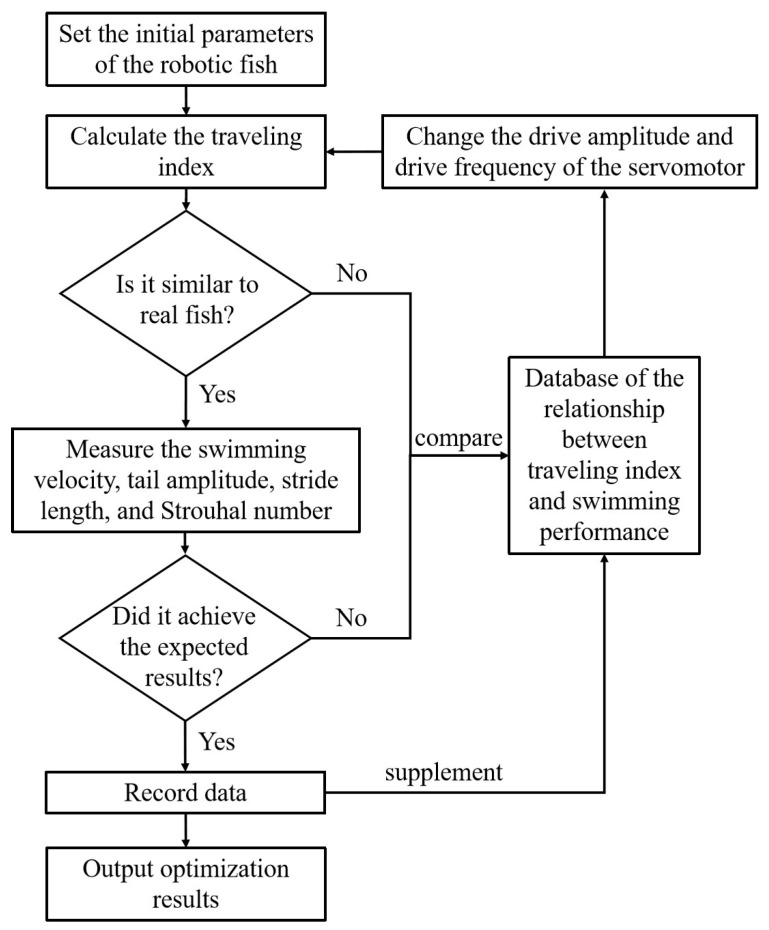
Strategy for optimization of the traveling index.

Experimental results show that optimizing the traveling index can substantially improve the swimming performance of the robotic fish. An optimization strategy is initially proposed, as shown in Figure 11, with the potential to realize iterative optimization of robotic fish. First, the initial parameters of the robotic fish are set, including structural size, software, and hardware design. Next, the COD method is used to calculate the traveling index of the robotic fish and compare it with real fish. If the traveling index is not similar to that of real fish, a database comparison of the relationship between traveling index and swimming performance is conducted. The amplitude and frequency of the driving servo motor change until the traveling index matches the biological data. The database records a large number of experimental results on the tensegrity robotic fish and can provide help in adjusting the traveling index. In the future, analysis of the database through machine learning methods may reveal deeper relationships between the traveling index and swimming performance. If the traveling index is similar to that of real fish, the swimming performance indicators are then measured, such as swimming velocity, tail amplitude, stride length, Strouhal number, etc. Next, we can determine whether these indicators meet expectations. If expectations are not satisfied, the steps outlined above are again repeated, referring to the database and adjusting the servo motor’s amplitude and frequency. If expectations are met, the experimental data are recorded, and the optimization results are output.

## 5. Conclusions

In this paper primarily studies the complex modal characteristics of a tensegrity robotic fish under vibration excitation. Initially, the fish body wave is decomposed into traveling and standing components using the complex orthogonal decomposition (COD) method. Their motion is quantitatively described using the traveling index. When the traveling index is high, the fish body wave is dominated by the traveling component, corresponding to the complex modal shape. Conversely, when the traveling index is low, the fish body wave is dominated by the standing component, corresponding to the real modal shape.

Experimental results indicate that the robotic fish exhibits complex modal shape body waves similar to those of real fish. Under various driving frequencies, the standing component of the robotic fish displays lower-order or higher-order vibration modes. Furthermore, the tensegrity robotic fish’s traveling index ranges from 0.4 to 0.7, which is close to the ranges of subcarangiform and carangiform fishes (0.52∼0.78).

We also preliminarily explored the relationship between traveling index and swimming performance. Experimental results indicate a high negative correlation between traveling index and swimming velocity, a low positive correlation with tail amplitude and stride length, and no correlation with Strouhal number. According to the proposed strategy for the optimization of the traveling index, it may be possible to further improve the swimming performance of the robotic fish after iterative optimization.

Investigating the relationship between the fish body waves and swimming performance is an important academic direction, such as exploring the high-frequency swimming performance of robotic fish [46], studying the body stiffness control mechanism [47], and investigating the morphology of fish [30]. The traveling index can quantitatively depict the relationship between the traveling and standing components. It could potentially serve as a crucial indicator to mimic the body waves of natural fish in robotic fish at the dynamic characteristic level. Different fishes have corresponding ranges of traveling index, whose deep connections with swimming performance, fish body stiffness properties, and fish morphology remain to be explored.

## Figures and Tables

**Table 1:** The correlation coefficients ( $R_{c}$ ) and *p* values between the swimming performance and the traveling indexes of the robotic fish.

Swimming Performance	Driving Amplitude (°)	The Correlation Coefficient $R_{c}$	*p* Value
Swimming velocity	36	−0.487	*p* < 0.01
	45	−0.717	*p* > 0.05
	54	−0.700	*p* < 0.01
	63	−0.463	*p* < 0.01
Tail amplitude	36	0.519	*p* < 0.01
	45	0.360	*p* < 0.01
	54	0.141	*p* < 0.01
	63	0.452	*p* < 0.01
Stride length	36	0.522	*p* < 0.01
	45	0.262	*p* < 0.01
	54	0.228	*p* < 0.01
	63	0.484	*p* < 0.01
Strouhal number	36	0.121	*p* < 0.05
	45	0.127	*p* > 0.05
	54	−0.274	*p* < 0.01
	63	0.059	*p* < 0.01

## Data Availability

The data that support the findings of this study are available from the corresponding authors upon reasonable request.
